# Editorial: Advances in molecular classification and targeting of solid tumors

**DOI:** 10.3389/fonc.2024.1373599

**Published:** 2024-02-13

**Authors:** Manoj Pandey, Tarun Kumar, Jill Koshiol

**Affiliations:** ^1^ Department of Surgical Oncology, Institute of Medical Sciences, Banaras Hindu University, Varanasi, India; ^2^ Infections and Immunoepidemiology Branch, Division of Cancer Epidemiology and Genetics (DCEG), National Cancer Institute (NCI), National Institutes of Health (NIH), Rockville, MD, United States

**Keywords:** molecular diagnosis, tumor target, precision medicine, precision oncology, personalized & precision medicine (PPM)

Increasing application of molecular techniques to diagnose solid tumors has led to improved understanding of the disease process, molecular classification, risk stratification, prognostication, and identification of newer therapeutic targets. For example, in 1962, Jansen (1, 2) described the estrogen receptor (ER) and its mechanism of action. The first human estrogen receptor (known today as ERα) was cloned in 1986 (3). Even before the cloning of the receptor, in 1978, Craig Jordon proposed a new way to treat human breast cancer using tamoxifen in a rat model (4). In 1971, Cole published early results of the use of tamoxifen in humans (5), and later this drug became available for palliative treatment of breast cancer. However, after the publication of the Early Breast Cancer Trialists’ Collaborative Group results, tamoxifen was accepted as adjuvant treatment for hormone receptor positive breast cancer (6). This use of tamoxifen represents the first therapeutic application of a molecular classification of cancer and the first example of how targeting a molecular mechanism can be effective for cancer treatment. From there onwards the last three decades have seen rapid development in molecular classification and increasing targeting of molecular mechanisms.

Innovations in molecular techniques like high-throughput sequencing and microarray techniques, coupled with advancements in computation and development of newer software, statistical techniques and machine learning, have contributed to the enrichment of classification. However, one of the most important advances was the discovery and availability of the reference human genome, without which all this classification would have been useless. It is only by aligning the patient data to the standard reference that one is able to identify single nucleotide polymorphisms and expression profiles. Use of fluorescent *in situ* hybridization (FISH) has led to the identification of translocations, and together these techniques have led to the discovery of many targets. Returning to the example of breast cancer, we now know that apart from the five cardinal molecular subtypes, that is luminal A, luminal B, Her2 enriched, normal breast and basal types, that are currently used for treatment, further types exist, such as claudin low, molecular apocrine, interferon related, immune enrich, and Luminal androgen receptor types. In addition, DNA repair deficiency and PI3K-mTOR pathway activated breast tumors are being explored to better understand how they might be targeted for treatment (7, 8).

While the technological advancement in diagnostics helped with the classification, the development in pharmacological techniques and drug development led to increased targeting. Identification of small molecule tyrosine kinase inhibitors, receptor targeting, monoclonal antibodies, drug delivery and gene delivery systems helped. Further advancements are expected as we develop mRNA vaccine techniques and gene editing techniques like CRISPR.

In light of these advances, we asked a number of experts in various solid organ tumors to share their perspectives in this Research Topic, leading to the six articles published in this Research Topic. Taghizadeh et al. presented the Austrian, tricentric, real-world, analysis of metastatic biliary tract tumors. They identified 205 molecular alterations, of which 198 were mutations that affected 89 genes in 61/92 patients. *KRAS*, *TP53*, *PIK3CA*, *FGFR2*, *IDH1*, *IDH2*, and *CDKN2A* were the main genes identified. Of these, the *PIK3-AKT* pathway, *FGFR*, *IDH*, and *CDKN2A* are targetable. In addition, *TP53* confers poor cancer prognosis. In this study, a total of 16 patients received targeted treatment. Sun et al. demonstrated the importance of public databases by reporting a bioinformatic study looking at the Mu opioid receptor mRNA in the data downloaded from CBioPortal. A significantly higher expression was found in patients with advanced T and M stage, and patients with higher expression had lower progression free and overall survival, suggesting Mu opioid receptors to be pan cancer prognostic markers.

Krauze et al. explored proteomic alterations in glioblastoma in 82 patients and identified 3 distinct clinical clusters with 389 significantly differentially expressed proteins. The three clinical groups had distinct survival profiles ranging from 13 months at the lowest to 28 months at the highest. Yang et al. investigated EPDR1 and tumour budding in 621 patients with bladder carcinoma. Higher expression of EPDR1 was seen with higher T, N and M stage and was associated with shorter survival of the patients. Xu et al. developed a novel prognostic model for gastric cancer prognostication using autophagy related genes. In their study they could identify two distinct clusters each having significant survival differences.

The last few years have seen great progress made in molecular diagnostic tools, allowing us to gain new perspectives on disease biology. Molecular classification has also helped to explain why different people react to the same treatment in different ways. The focus is now on identifying molecular signatures of distinct tumors, as we have seen in the articles highlighted through this Research Topic. These molecular signatures could lead to the discovery of specific mutations that might be used as prognostic indicators and therapeutic targets for different medications. The novel targets identified through these studies should accelerate research into developing more targeted treatments.

## Author contributions

MP: Conceptualization, Supervision, Writing – original draft, Writing – review & editing. TK: Formal analysis, Project administration, Resources, Writing – review & editing. JK: Conceptualization, Funding acquisition, Investigation, Methodology, Resources, Writing – original draft, Writing – review & editing.
